# The Safety and Efficacy of Intra-Arterial versus Intravenous Neoadjuvant Chemotherapy in Patients with Locally Advanced Cervical Cancer: A Meta-Analysis

**DOI:** 10.1155/2020/5023405

**Published:** 2020-02-25

**Authors:** Cheng Liu, Ran Cui, Miaomiao Li, Ying Feng, Huimin Bai, Zhenyu Zhang

**Affiliations:** Department of Obstetrics and Gynecology, Beijing Chao-Yang Hospital, Capital Medical University, Beijing, China

## Abstract

**Objective:**

The aim of this study was to evaluate the safety and efficacy of intra-arterial versus intravenous neoadjuvant chemotherapy for the management of patients with locally advanced cervical cancer.

**Methods:**

The PubMed, EMBASE, PMC, Web of Science, and Cochrane databases were searched to identify correlational studies published in English. Prospective controlled studies that evaluated the treatment effect of intra-arterial neoadjuvant chemotherapy or intravenous neoadjuvant chemotherapy in patients with locally advanced cervical cancer were pooled for a meta-analysis.

**Results:**

A total of three eligible studies with 112 patients with locally advanced cervical cancer were eventually included in this analysis. The baseline regimen of neoadjuvant chemotherapy was platinum-based chemotherapy. The total clinical response rate was 71.4%, and the overall pathological complete response (CR) rate was 11.5%. The grade 3/4 toxicity rate was 27.2%. In the intra-arterial group, the response rate was 83.1% (CR, 22.0%; partial response (PR), 61.0%), which was significantly higher than 58.5% (CR, 11.3%; PR, 47.2%) in the intravenous group (*P*=0.01). The pathological CR rate was 15.5% in the intra-arterial group, which was higher than 6.5% in the intravenous group. The grade 3/4 toxicity rate was 17.2% in the intra-arterial group, which was higher than the rate of 13.8% in the intravenous group.

**Conclusion:**

Platinum-based neoadjuvant chemotherapy was well tolerated in patients with locally advanced cervical cancer and showed moderate response activity. Compared to intravenous neoadjuvant chemotherapy, intra-arterial neoadjuvant chemotherapy had an evident advantage in terms of the clinical response while maintaining a similar toxicity rate. The clinical efficacy of intra-arterial neoadjuvant chemotherapy deserves further evaluation.

## 1. Introduction

Cervical cancer is the third most common cancer in women worldwide [1]. With improvements in screening technology, the incidence of cervical cancer has decreased [2]. However, this tumour type is still one of the most frequent causes of death from malignant diseases in women in developing countries, and a large number of patients present with locally advanced cervical cancer, that is, with 2009 International Federation of Gynecology and Obstetrics (FIGO) stages IB2–IVA at the time of diagnosis [3]. Bulky tumours in the cervix are a significant negative prognostic predictor in patients with locally advanced cervical cancer [4].

Neoadjuvant chemotherapy, including intravenous or intra-arterial neoadjuvant chemotherapy, prior to radical hysterectomy, can be an alternative treatment method for patients with locally advanced cervical cancer. However, the exact role of preoperative therapy in the treatment of cervical cancer remains controversial. Several studies have proposed that neoadjuvant chemotherapy could effectively improve pelvic control and eradicate distant micro-metastasis of bulky tumours [5–8]. The effect of chemotherapy might be affected by the routes of drug administration. Some studies have reported that intra-arterial neoadjuvant chemotherapy is associated with a higher clinical response rate than intravenous chemotherapy, thus resulting in a more favourable prognosis [9–12]. However, all of these studies have several limitations, such as small sample sizes, no strict control groups, and retrospective designs. A definitive conclusion cannot be drawn based on the current published data. Consequently, a meta-analysis was performed using pooled data from previously published prospective studies. We compared the treatment effects of intra-arterial and intravenous neoadjuvant chemotherapy to provide information that could be used to improve the clinical outcomes of patients with locally advanced cervical cancer.

## 2. Methods

### 2.1. Search Strategy

Relevant publications were identified by conducting a literature search in the PubMed, PMC, EMBASE, Web of Science, and Cochrane databases based on the following medical subject headings: “cervical cancer,” “neoadjuvant chemotherapy,” “intravenous,” and “intra-arterial”. For example, the search was performed using the following Boolean search: “cervical cancer” AND (“neoadjuvant chemotherapy” OR (“intra-arterial” or “intravenous”)). Cross-referencing of the retrieved articles was also performed to identify any additional data that could be included in the meta-analysis.

### 2.2. Inclusion Criteria

Candidate studies were pooled for analysis based on the following inclusion criteria: (1) original studies; (2) patients with locally advanced cervical cancer; (3) staging based on the 2009 FIGO system; (4) patients who received no chemotherapy or radiotherapy prior to the clinical trial; (5) cohort or case-control studies that evaluated the safety and/or efficacy of intra-arterial vs. intravenous neoadjuvant chemotherapy; (6) sufficient original data were provided to estimate the treatment effect odds ratios and corresponding 95% confidence intervals (95% CIs); and (7) patients received radical hysterectomy after neoadjuvant chemotherapy without surgical contraindications. The exclusion criteria included the following: (1) review studies or isolated case reports; (2) duplicate studies; (3) missing ethics board approval; (4) studies without full-text articles or incomplete data; and (5) studies not written in English.

### 2.3. Data Extraction

According to the PRISMA guidelines, the initial review of the titles and abstracts of the candidate studies was independently conducted by two researchers (Liu and Bai). Data from the eligible articles and subsequent data from other references were reviewed independently. The following baseline information was collected: title; name of the first author; year of publication; journal; inclusion year(s); country or region of origin; patient ethnicity; study design; patient age at diagnosis; cancer clinical stage; numbers of patients and controls; clinical response to chemotherapy; toxicity; follow-up period; disease recurrence; and morbidity. The objective responses of the patients and tumour operability were reappraised based on a second clinical examination and repeated abdominopelvic magnetic resonance imaging. Complete response (CR) was defined as the disappearance of any measurable disease. Partial response (PR), stable disease (SD), and progressive disease (PD) were defined as a 50% or greater reduction, a reduction < 50% or an increase < 25%, and a 25% or greater increase in the product of the transverse diameters of the cervical lesions, respectively. The total clinical response rate included both CR and PR. Pathological CR was defined as no residual disease on the pathological examination. The toxicity assessment was performed according to the National Cancer Institute Common Toxicity Criteria version 3. Data regarding high-risk factors for recurrence and death, such as a large tumour diameter (>4 cm), parametrial infiltration, lymph node metastasis, and intraoperative and postoperative complications, were collected and evaluated. For the relevant data that were reported only graphically, values were estimated from the graphs. Progression-free survival was calculated from the date of diagnosis to the time of recurrence; women living free of disease at the time of their last contact were censored. Overall survival was calculated from the date of diagnosis to the date of death; women who were still alive at the time of their last contact were censored.

### 2.4. Analytical Approach

The data analysis and bias risk assessment were performed with RevMan 5.3 software from the Cochrane Collaboration. Heterogeneity among studies was determined using the *Q*-test and the *I*_2_ test. When *P* < 0.05 for the *Q*-test or *I*_2_ < 50%, indicating the absence of heterogeneity, a fixed-effects model was used to estimate the pooled overall rates and 95% CIs. Otherwise, a random-effects model was applied. Chi-square heterogeneity tests were used to test gross statistical heterogeneity across trials, and chi-square tests of interactions or trends were used to test the differences in outcome heterogeneity between subsets of trials or between subgroups of patients. Sensitivity analyses were performed to assess the stability of the pooled results. Funnel plots were used to investigate publication bias. The survival rate of the patients was calculated according to the Kaplan–Meier method. All *P* values were two-tailed unless otherwise stated. *P* < 0.05 was considered statistically significant.

## 3. Results

**Figure 1:**
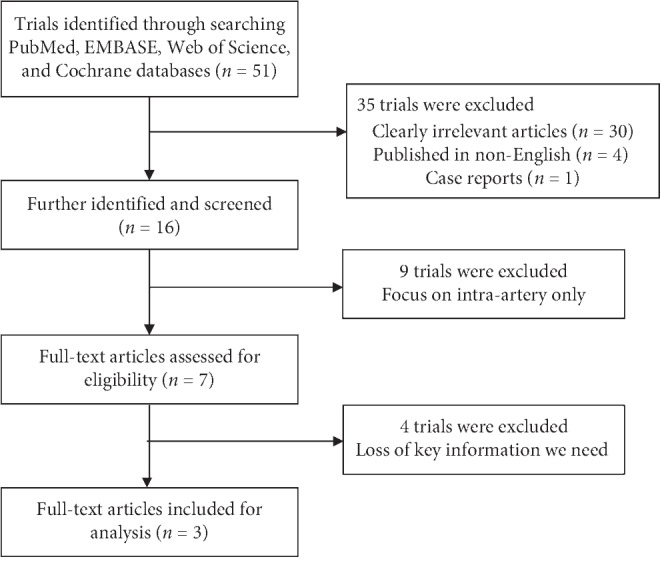
Flowchart of literature search and study selection.

**Figure 2:**
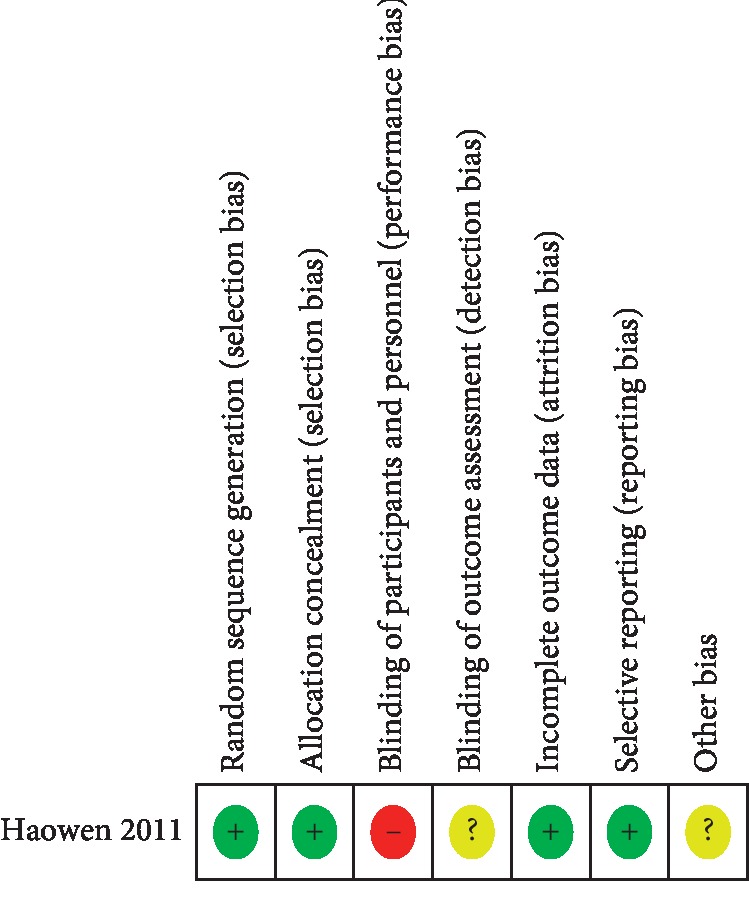
Risk of bias summary for randomized controlled trial conducted by Wen et al.

A total of 51 nonduplicated studies were identified from the databases by searching with the medical subject heading terms between October 1996 and May 2018. Thirty studies were excluded after reviewing the titles and abstracts and determining that the studies contained irrelevant information. One isolated case report was also excluded. Nine studies focusing on either intra-arterial or intravenous neoadjuvant chemotherapy without a control group were further excluded. Four studies lacking information about the particular clinical response, toxicity, or other necessary data were also excluded. Thus, a total of three prospective controlled studies with 112 patients were eventually included in this analysis (Figure 1) [10–12]. The Newcastle Ottawa Scale assessment [13] showed that the quality of the two included cohort studies [11, 12] was excellent (6–8 points). A randomized clinical trial conducted by Wen and his colleagues [10] had a low risk of bias for the following areas: blinding of participants and personnel, allocation concealment, blinding of outcome assessment, and incomplete outcome data (Figure 2).

The study design and clinicopathological characteristics of the patients in the available eligible trials are shown in Tables 1 and 2. The average age of the patients at diagnosis was 45.6 years. The most common histology types were squamous cell carcinoma (73 cases), adenocarcinoma/adenosquamous carcinoma (36 cases), and undifferentiated carcinoma (3 cases). The FIGO stage ranged from IB-IIIB. Neoadjuvant chemotherapy was performed in all patients, and the regimens included cisplatin with 5-fluorouracil or aclacinomycin A. Intra-arterial and intravenous neoadjuvant chemotherapy were performed in 59 (52.7%) and 53 (47.3%) patients, respectively. Patient responses to chemotherapy and the feasibility of surgery were evaluated two to four weeks after the end of the second cycle of neoadjuvant chemotherapy. For those who did not achieve a sufficient response (CR or PR), a third cycle of chemotherapy was performed (13 patients, 11.6%). Radiotherapy was performed in 32 patients (28.6%). The total clinical response rate was 71.4%, and the pathological CR rate was 11.5%. The grade 3/4 toxicity rate was 27.2% and included neutropenia, anaemia, thrombocytopenia, nausea, and diarrhoea. Nutritional support and symptomatic treatment were administered. Due to persistent and serious toxicity, two patients, one in the intravenous group for continuous diarrhoea and one in the intra-arterial group for severe neutropenia and thrombocytopenia, received additional radiotherapy and/or chemotherapy instead of radical hysterectomy. No neoadjuvant chemotherapy-related death was reported. For unspecified reasons, nine patients received additional radiotherapy and/or chemotherapy, and two patients refused any further treatment. Thus, the scheduled radical hysterectomy was performed in 99 operable patients.

**Figure 3:**
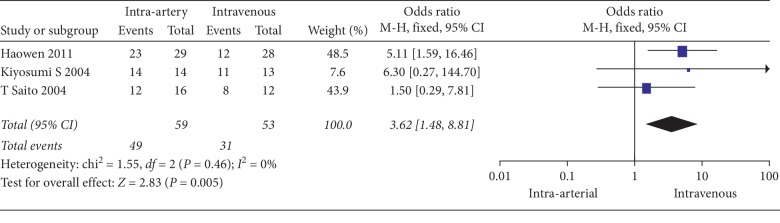
Forest plot of the response rate between intra-arterial and intravenous chemotherapy.

**Figure 4:**
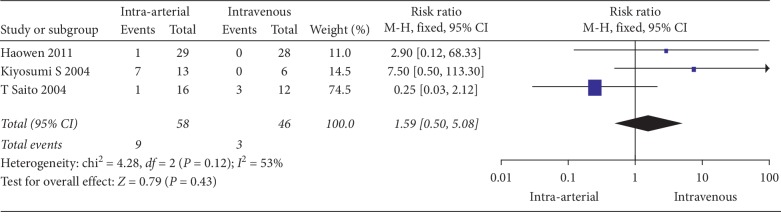
Forest plot of the pathological completed response rate between intra-arterial chemotherapy.

The clinical response of patients treated with intra-arterial and intravenous neoadjuvant chemotherapy is compared in Table 3. A fixed-effects model was applied for the comparison of the response rate due to low heterogeneity (*I*_2_ = 0%, *P*=0.46). A random-effects model was used for the comparison of histological effects due to substantial heterogeneity across the pooled studies (*I*_2_ = 53%, *P*=0.12). The response rate of patients who received intra-arterial neoadjuvant chemotherapy was 83.1%, which was significantly higher than the 58.5% response rate of patients who were treated with intravenous neoadjuvant chemotherapy (OR = 3.62, 95% CI: 1.48 to 8.81, *P*=0.01, Figure 3). A total of nine (15.5%) patients achieved pathological CR in the intra-arterial group, which was higher than the 6.5% of patients in the intravenous group, although the result was not statistically significant (*P*=0.43, Figure 4). The results of the *Q*-test did not reveal evident heterogeneity (*P*=0.12), but the *I*_2_ test, which is more sensitive, showed substantial heterogeneity (*I*_2_ = 53%, Figure 4). To explore the source of heterogeneity, a sensitivity analysis was performed by eliminating the included studies one by one, and eventually, the pooled results also showed no significant changes. No severe intraoperative or postoperative complications were reported in either of the groups.

## 4. Discussion

Chemoradiotherapy is considered by many academic organizations to be the standard treatment for locally advanced cervical cancer [14]. This procedure includes pelvic external-beam radiotherapy with concomitant platinum-based chemotherapy followed by brachytherapy to boost the central disease response. However, multiple serious side effects, such as inflammatory bowel disease, acute or chronic radiation cystitis, and vaginal stenosis, negatively affect the quality of life of patients [15]. Owing to its positive psychological impact on remission, most patients prefer to undergo tumour resection. As a timely and efficient treatment, neoadjuvant chemotherapy may be preferred by women when waiting for the proper opportunity for operation and by surgeons because of its significant effect on tumour reduction [16].

Neoadjuvant chemotherapy can significantly reduce the tumour size in the cervix, which is a significant prognostic factor because bulky tumours are associated with a higher risk of lymph node metastases and recurrence than smaller tumours [17, 18]. Neoadjuvant chemotherapy prior to surgery may reduce the tumour size, rendering inoperable tumours operable and controlling micrometastatic disease [19]. A systematic review and meta-analysis of five randomized clinical trials included 872 patients with locally advanced cervical cancer and showed that compared to radiotherapy alone, neoadjuvant chemotherapy and radical hysterectomy (with or without postoperative radiotherapy) significantly improved the two-year and five-year survival rates of this patient group by 8% to 14% and 12% to 16%, respectively [16]. However, a randomized clinical trial published in 2007 demonstrated that neoadjuvant chemotherapy offered no additional objective benefit to patients with stage IB cervical cancer who underwent radical hysterectomy and pelvic/para-aortic lymphadenectomy [20]. The five-year disease-free survival rate was worse in patients who received neoadjuvant chemotherapy than in those who received chemoradiotherapy [21]. Nevertheless, these results still need further evaluation due to their heterogeneous chemotherapeutic regimens and administration routes. The studies included in this analysis focused exclusively on platinum-based regimens and had similar trial designs. The tumour stage in these three studies was based on the 1994 FIGO staging system. However, we still used the 2009 FIGO staging system as our inclusion criterion because there is no difference between these two staging systems when defining locally advanced cervical cancer, which was the focus of our study. Based on our data, the three-year survival rate was 78.8% for patients with locally advanced cervical cancer who received neoadjuvant chemotherapy, which is similar to the results obtained in other studies.

However, via the intra-arterial approach, cytotoxic agents can be carried directly to the target tumours and have a more immediate effect on uterine and para-uterine lesions. In addition, intra-arterial neoadjuvant chemotherapy results in a high clinical response rate, which is a reliable predictor of a good prognosis and a long survival period [22]. Several previous studies have shown that the clinical response rate of locally advanced cervical cancer patients was 52% to 91% with the use of various platinum-based intravenous neoadjuvant chemotherapy regimens [20, 23, 24]. Similar results were also reported in studies of platinum-based intra-arterial neoadjuvant chemotherapy, with an overall response rate of 63.6% to 91.7% [25–27]. Our analysis showed that the total clinical response rate was 71.4%, with rates of 83.1% and 58.5% in patients treated with intra-arterial and intravenous neoadjuvant chemotherapy, respectively. The total clinical response rate with intra-arterial treatment reported in the current study was higher than the average rate reported in other studies, while that with intravenous treatment was lower. However, few studies have compared the differences between these two approaches. Our data showed that intra-arterial neoadjuvant chemotherapy resulted in a significantly better clinical response than did intravenous chemotherapy, suggesting a potential survival benefit associated with intra-arterial chemotherapy.

Intra-arterial infusion may lead to a stronger antitumour effect on primary tumours than intravenous infusion, possibly because of the higher drug concentration achieved in the tumour tissue with intra-arterial infusion and the high tissue binding affinity of platinum [28, 29]. Animal studies have shown higher rates of drug infiltration and a higher concentration of the drug in the target tumour lesion with intra-arterial infusion compared with intravenous drug infusion [30, 31]. Cancer cell metastasis is similar to cell homing in tumour lesions and cell engraftment in remote tissues [32]. Recently, published series have confirmed that an optimal pathological response is a strong predictor of survival in patients with locally advanced cervical cancer [33]. In our analysis, intra-arterial neoadjuvant chemotherapy resulted in a better pathological CR than did intravenous chemotherapy, as well as a better outcome with regard to lymph node metastasis, which may also suggest the superiority of intra-arterial infusion for the improvement of the prognosis of patients with locally advanced cervical cancer. In addition, a recent pooled analysis [34] showed that achieving a complete pathological response to neoadjuvant chemotherapy improves both event-free survival and overall survival in patients with breast cancer. These data indicate that a good pathological response may be an important predictor of prognosis and that intra-arterial neoadjuvant chemotherapy can lead to a better prognosis due to its benefits with regard to pathological improvements in patients with locally advanced cervical cancer.

Regarding the safety of neoadjuvant chemotherapy, the total grade 3/4 toxicity rate was 27.2%. The main toxic effects of neoadjuvant chemotherapy in our eligible studies were neutropenia, anaemia, and thrombocytopenia. However, most of these effects were temporary, and most patients recovered quickly after treatment. Only two patients could not undergo surgery due to persistent toxicity related to neoadjuvant chemotherapy. No treatment-related deaths occurred. Therefore, neoadjuvant chemotherapy was well tolerated in patients with locally advanced cervical cancer, and the incidence of toxicity was not significant.

### 4.1. Strengths and Limitations

The eligible studies were all prospective randomized clinical trial studies and provided the necessary data, which indicates that the conclusions drawn in this meta-analysis are relatively credible. The major limitation of this meta-analysis was the small number of eligible patients. In addition, despite our efforts to perform a comprehensive search of the literature, potential publication bias could not be ruled out because papers with positive results are more likely to be published.

In conclusion, neoadjuvant chemotherapy was well tolerated and showed moderate activity in locally advanced cervical cancer. Compared to intravenous neoadjuvant chemotherapy, intra-arterial neoadjuvant chemotherapy has evident advantages in terms of the clinical response while maintaining similar toxicity rates. The clinical efficacy of intra-arterial neoadjuvant chemotherapy deserves further evaluation.

## Figures and Tables

**Table 1:** Study design of eligible studies.

Study	Study type	Stage	Age (average)	NACT regimen	Duration of NACT	Safety assessment
Saito et al. [11]	Prospective cohort study	IB-IIIB	28–75 (45.4)	CDDP 70 mg/m2 ACM 30 mg/m2 MMC 5 mg/m2	Every 14 days for two cycles	HT and NHT
Shibata et al. [12]	Prospective cohort study	IB2-IIIB	29–73 (46.5)	5-FU 700 mg/m2 5-FU 700 mg/m2	Every 22 days for two cycles	HT and NHT
Wen et al. [10]	RCT	IB2-IIA	17–75 (44.8)	CDDP 50 mg/m2 5-FU 700 mg/m2	Every 14 days for two cycles	HT and NHT

RCT, randomized controlled trial; CDDP, cisplatin; ACM, aciacinomycin; MMC, mitomycin; 5-FU, 5-fluorouracil; HT, hematological toxicities; NHT, nonhematological toxicities.

**Table 2:** Patient baseline characteristics.

Study	Patients enrolled	Histology type			Lymph node			Survival endpoints
		SCC	Adeno	Undifferentiated	Positive	Negative	Unknown	
Saito et al. [11]	28	0	26	2	12	11	5	5-year survival 87.5%
Shibata et al. [12]	27	20	6	1	2	15	2	Disease-free survival 85.2%
Wen et al. [10]	57	53	4	0	12	16	29	3-yeat FPS 80.7% 3-year OS 80.7%

SCC, squamous cell carcinoma; Adeno, adenocarcinoma; FPS, progression-free; OS, overall survival.

**Table 3:** Efficacy response endpoint.

Chemotherapy approach	No. of patients enrolled	RR	CR	PR	PD	SD	PCR
Intra-arterial	59	49	13	36	4	6	9
Intravenous	53	31	6	25	6	16	3

RR, response rate; CR, complete response; PR, partial response; PD, progressive disease; PCR, pathological complete response.
